# Prevalence and factors associated with the use of traditional medicine in individuals with hypercholesterolemia, hyperglycaemia, and arterial hypertension in Ecuador: results from a population-based study in two health districts

**DOI:** 10.1186/s12906-024-04666-0

**Published:** 2024-10-07

**Authors:** Marta Puig-García, Carmen López-Herraiz, Cintia Caicedo-Montaño, María Fernanda Rivadeneira, Juan Vásconez-Donoso, Gregorio Montalvo-Villacis, Ikram Benazizi-Dahbi, Lucy Anne Parker, CEAD group, Ana Lucía Torres Castillo, Andrés Peralta, Elisa Chilet-Rosell, Francisco Barrera Guarderas, Jessica Pinto Delgado, María Hernández, Mónica Marquez-Figueroa, Sergio Morales-Garzón

**Affiliations:** 1 Department of Public Health, Universidad Miguel Hernández de Elche, Alicante, Spain; 2 CIBER de Epidemiología y Salud Pública (CIBERESP), Madrid, Spain; 3 Centre of Community Epidemiology and Tropical Medicine (CECOMET), Esmeraldas, Ecuador; 4 Institute of Public Health, Faculty of Medicine, Pontificia Universidad Católica del Ecuador, Quito, Ecuador; 5 Faculty of Nursing, Pontificia Universidad Católica del Ecuador, Quito, Ecuador; 6 School of Medical Specialities, Colegio de Ciencias de la Salud, Universidad San Francisco de Quito, Quito, Ecuador

**Keywords:** Traditional medicine, Cardiovascular diseases, Ecuador, Observational study, Pharmacoepidemiology

## Abstract

**Background:**

While traditional medicine (TM) is employed by a significant portion of the global population for managing health issues, clinical guidelines and state recommendations often overlook this practice. The aim of this study was to describe the frequency of use of TM to control 3 metabolic risk factors (MRF): hypertension, hypercholesterolemia, and hyperglycaemia; and the sociodemographic, economic, and clinical characteristics associated with the use of TM.

**Methods:**

Cross-sectional descriptive study that analyses data obtained from a representative population survey in 2 health districts, one urban in the south of Quito and another in a forested rural area with diverse ethnic groups in Esmeraldas, Ecuador. We include 602 individuals with at least one MRF. We calculated the proportion of people reporting the regular use of TM (herbal or traditional remedy) to control their MRF and we assessed potential associations with sociodemographic, economic, and clinical characteristics with a multivariable logistic regression model.

**Results:**

In two very different sociocultural contexts in Ecuador we found that use of TM to control MRF was frequent (39.4% in Esmeraldas, 31.1% in Quito), frequently in combination with CM. There is a notable percentage of people, 33.9% in Esmeraldas and 39.0% in Quito, who did not take any treatment for their MRF, and the remainder used CM alone. In both settings, an individual’s education lever was significantly associated with TM use. Whereas in Quito individuals with higher education more frequently treated their MRF with TM (aOR 2.04, 95% CI 1.03–3.90), in the rural, hard-to-reach context of Esmeraldas, it was more frequent among people with no formal schooling (aOR: 3.76; 95%CI 1.59–8.88), as well as those of younger age (aOR by year: 0.97; 95% CI 0.95–0.99) and afro ethnicity (aOR: 2.13; 95%CI 1.02–4.45).

**Conclusion:**

Traditional medicine is used by a significant proportion of the population in Ecuador, highlighting the need for a more accessible and intercultural healthcare approach. The health system should ensure access to the necessary information and resources for the management of their metabolic risk factors.

**Supplementary Information:**

The online version contains supplementary material available at 10.1186/s12906-024-04666-0.

## Background

 Non-communicable diseases (NCDs) account for 74% of global mortality, with 77% of these occurrences occurring in low- and middle-income countries [1]. In Ecuador, where this study takes place, 58% of deaths in 2021 were attributed to NCDs, with cardiovascular diseases (CVD) standing out at 24% of total deaths in the country [2]. In 2022, ischemic heart disease was the primary cause of mortality in Ecuador, followed by diabetes mellitus and cerebrovascular disease [3].

An unhealthy diet, physical inactivity, and tobacco and alcohol consumption can manifest as metabolic risk factors such as hypertension, hypercholesterolemia, and hyperglycaemia [4]. Numerous studies have demonstrated the association between various risk factors and CVD, highlighting that controlling these factors leads to a reduction in mortality [4, 5]. However, the increasing number of people with cardiovascular diseases is also closely linked to changes in demographic patterns in impoverished countries influenced by globalization, market liberalization, and massive urbanization. These changes result in a population shift towards progressively less healthy “modifiable” health behaviours [6, 7]. Furthermore, this shift is occurring in contexts lacking adequate access to healthcare and pharmacological treatment.

In Ecuador, the healthcare system is fragmented and access depends on geographic accessibility and economic resources, manifesting significant disparities between urban and rural areas. Healthcare facilities within the National Health System (NHS) include establishments overseen by the Ministry of Public Health as the governing body of the NHS, with a predominant presence at the primary care level, alongside establishments within a social security system (for working individuals), military and police health facilities as well as a private clinics and practices. The fragmented nature of the system, together with issues related to medication shortages have led to significant out-of-pocket expenses, especially during vulnerable times such as the COVID-19 pandemic. In this context, an increasing use of traditional medicine (TM) can be observed as a measure to counteract the limitations of the conventional healthcare system [8].

The World Health Organization (WHO) defines traditional medicine as the sum total of the knowledge, skills, and practices based on the theories, beliefs, and experiences indigenous to different cultures, whether explainable or not, used to maintain health and prevent, diagnose, improve, or treat physical and mental illnesses [9]. TM has been used for centuries to treat various pathologies and is considered in many countries to be an integral part of their cultural heritage and healthcare system [10, 11].

In Ecuador, the Constitution of the Republic of 2008, in its Article 32, mandates a health system with universal, open access, based on a family and community care model [12]. Similarly, Article 363 ensures the practices of ancestral and alternative healthcare through the recognition, respect, and promotion of the use of their knowledge, medicines, and instruments. This is achieved through the formulation, coordination, and implementation of public policies, plans, and programs with intercultural relevance within the NHS, ensuring access, recognition, and respect for the diversity of peoples and nationalities. Additionally, it strengthens, articulates, and incorporates ancestral-traditional medicine and alternative-complementary medicine, establishing within the Ministry of Public Health the National Directorate of Intercultural Health and Equity for the regulation, control, and oversight of their application.

Complementary to these initiatives, the Organic Health Law of 2006, Chapter II, Article 26, stipulates the promotion and development of traditional, ancestral, and alternative medicine [13]. In addition, the 2018 Manual of the Comprehensive Care Model of the National Family and Community Health System (MAIS-FCI) emphasizes the importance of interculturality and addresses the existing diversity between the indigenous population and health services. These legal frameworks and policies reflect the Ecuadorian state’s commitment to inclusive healthcare tailored to the diverse cultural needs of its population.

However, despite the intention to achieve an Intercultural Health System, integrating traditional practices into the conventional healthcare system faces challenges [14, 15]. This is primarily due to healthcare professionals’ reservations about patient safety when using natural remedies [16]. Concerns about therapeutic interactions, side effects, lack of studies, unregulated products, and the associated risk of adulteration are some of the reasons for reluctance towards TM. The most common factor contributing to this hesitancy is the lack of scientific evidence [9, 17].

Despite advances in the field of ethnopharmacy, there is a lack of scientific studies investigating the effectiveness of pharmacological compounds in medicinal plants, which are still widely used by a significant part of the population [18, 19]. While consumption of TM is more prevalent in rural areas, largely due to the higher proportion of indigenous and local populations, who rely on TM as their primary source of healthcare, based on inherited knowledge of herbal remedies [20, 21], the use of TM has spread to the urban population [22].

Traditional medicine is closely linked to ancestral knowledge and popular culture, with a strong connection to religious and spiritual beliefs based on a social construction of the health-illness process which collides with the scientific positivist ideals of conventional medicine [23]. This misalignment can lead to situations of discrimination in clinical settings where healthcare professionals may not have the training necessary to integrate these forms of knowledge into conventional medicine [24].

The choice of treatment for a particular condition depends primarily on personal knowledge and experience, which determines the preference for conventional or traditional medicine [21, 25]. However, in many cases, socio-economic barriers and inequities in access to the healthcare system determine the choice of treatment, with TM considered more affordable and readily available [23, 25, 26]. In fact, the WHO estimated that 80% of rural populations in developing countries rely on TM for maintaining their health [27]. Currently, there is a concurrent use of conventional and traditional medicine [21, 24], emphasizing the importance of providing comprehensive and intercultural care for the well-being of individuals.

Given this situation, it is crucial to provide information about how the population manages their risk factors with TM. This data can pave the way for future research and the development of prevention proposals for CVD that are more tailored to the needs and characteristics of the population.

## Methods

### Aim

To describe the frequency of TM in the control of 3 metabolic risk factors (hypertension, hypercholesterolemia, and hyperglycaemia) and explore the sociodemographic, economic, and clinical characteristics associated with the use of TM.

### Study design and setting

We undertook a representative cross-sectional study based on a population survey in two territorial health districts (the basic unit of planning and provision of public health services) of Ecuador; an urban health district in the south of Quito and a rural health district in the province of Esmeraldas. In Quito, data was collected by a survey team previously trained and supervised by the research team. In the rural area of Esmeraldas, a team with healthcare training undertook the survey under the supervision of the Centre of Community Epidemiology and Tropical Medicine (CECOMET) research staff with the support of local health promoters of each community to facilitate the survey process. Both survey teams were gender-balanced and collected the data during different times; Quito between March and October 2021, and Esmeraldas between August 2020 and January 2022. The questionnaire used in the population survey included sections from the WHO STEPS Non-Communicable Disease Risk Factors Survey forms [28]. The complete description of the methodology, including the sample size calculation, can be found in the study protocol [29].

#### Sampling strategy

In Quito, we employed a multistage cluster sampling strategy. In the first phase, we randomly selected 60 out of 1,024 census tracts in the health district, using a selection probability based on population size. In the second phase, we created 20 GPS points using QGIS within the urbanised areas of each selected census tract − 12 primary and 8 possible substitute points. If the selected person was unavailable, up to three rescheduled visits were attempted; otherwise, the GPS point was replaced. In the third phase, we went to the building that was closest to each GPS point, randomly selected one household within multi-household buildings, and invited one person aged 18 years or older from each household to participate. We invited 996 individuals, of whom 656 (65.9%) completed the survey.

In Esmeraldas, the sampling approach changed based on the setting. For urban centres (Borbón and Limones), we used a geospatial sampling similar to Quito generating 328 GPS points. For rural communities, we employed multistage stratified cluster sampling. We stratified communities according to ethnicity (afro-Ecuadorian, indigenous, mestizo) and isolation (distance and time from urban areas). We selected 60 community clusters, with the number of clusters in each stratum proportionate to the estimated population size. From each selected cluster, 8 participants were randomly selected, using a census updated by CECOMET and local health promoters between November 2018 and January 2020. We invited 628 individuals from these rural communities, of whom 490 (78%) completed the survey. In the urban communities of Borbón and Limones, 295 participants were invited, with 241 (81%) completing the survey. This resulted in a total sample of 731 participants from Esmeraldas.

### Participants

**Fig. 1:**
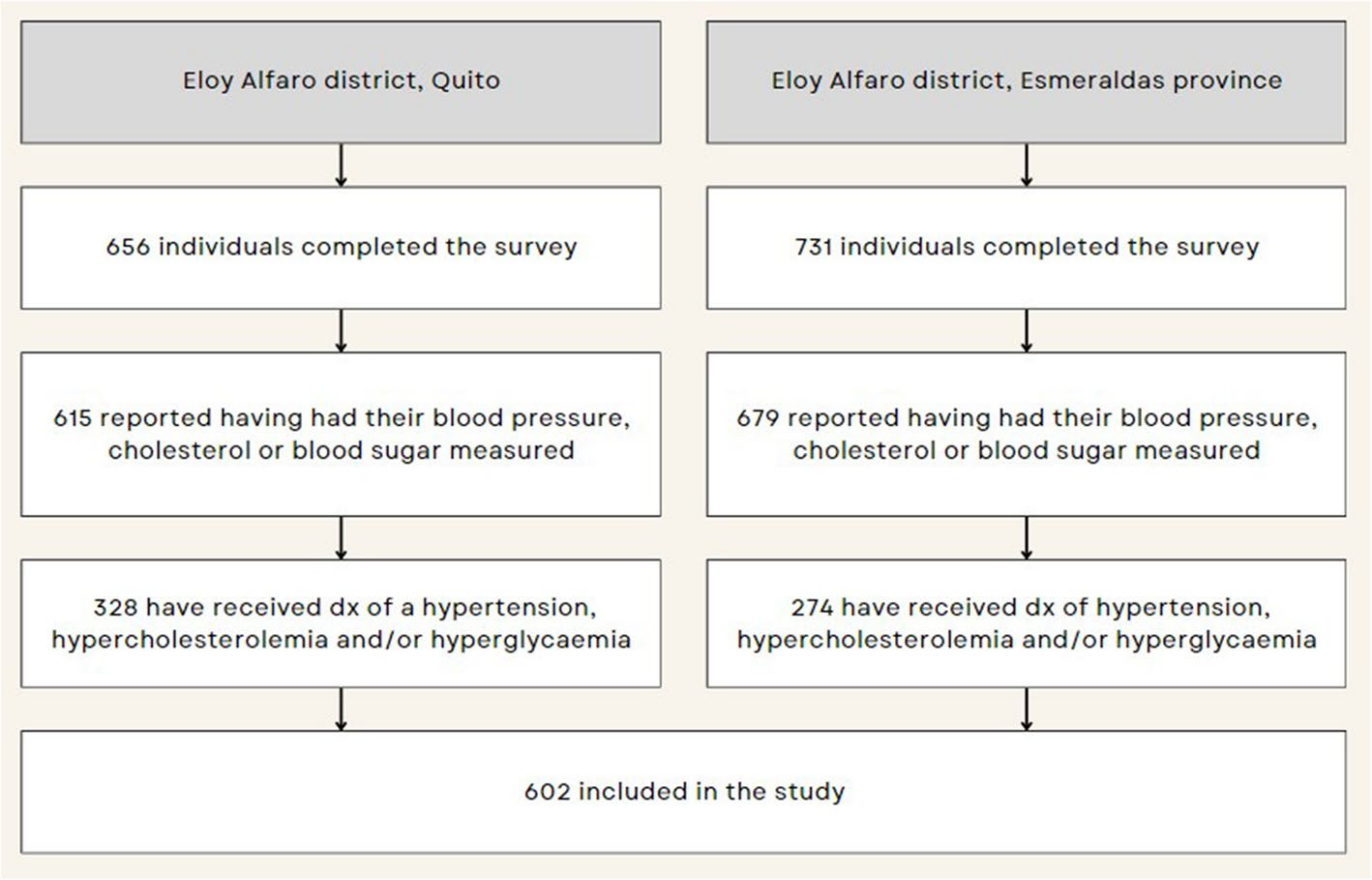
Participant flow diagram of Quito and Esmeraldas

For this descriptive study, we restricted our analysis to all individuals who reported having been diagnosed with hyperglycaemia, hypertension, and/or hypercholesteromia by a doctor or health worker previous to the survey (Have you ever been told by a doctor or other health worker that you have hypertension/diabetes/raised total cholesterol?). The specific questions from the STEPS survey can be found in Additional file 1. We obtained a final sample of 602 participants (328 in Quito and 274 in Esmeraldas) (Fig. 1).

### Variables

Regarding the sociodemographic, economic, and clinical characteristics of the participants, we collected data on place of residence, sex, age, employment status, educational level, income, ethnicity, and the number of metabolic risk factors (MRF), which include hyperglycaemia, hypertension and hypercholesterolemia. Participants from the Esmeraldas region were classified by their place of residence as either urban (for those living in Limones or Borbón) or rural (for those living in the smaller communities along the Santiago, Cayapas and Onzole rivers). This classification accounted for the distinct differences in accessibility and services between the more connected urban centres and the isolated rural communities, which are situated in dense forest and accessible primarily by boat. Age was considered as a continuous variable. We categorised some variables to facilitate the analysis and interpretation. Self-defined ethnicity was simplified to Afro-Ecuadorian, Mestizo, Indigenous and White. We classified completed education into four levels according to the education system is organised in Ecuador (no formal schooling, primary school, secondary school and higher education), due to low frequencies in higher education we combined it with secondary education. Marital status was classified into two categories: partnered (married or free union) or unpartnered (single, separated or widow). Employment status was dichotomised into formal employment (including self-employed, private sector employee or government employee) or not in formal employment (homemakers, students, unemployed or retired) due to the low frequencies in some categories. The item for estimated household earnings was stratified in Quito according to the Basic Monthly Salary in Ecuador when the study was carried out ($0 to $375 and over $375). Given the significantly overall lower earnings in Esmeraldas, a region classified as poor and extremely poor due to high levels of unsatisfied basic needs [30], we decided to use the median to reflect local economy conditions ($0 to $100 and over $100).

We calculated the number of MRF according to the diagnosis of hyperglycaemia, hypertension and/or hypercholesterolemia. The variable setting, dichotomised as urban or rural, related only to Esmeraldas. We created dichotomous variables (yes/no) for the use of traditional medicine (TM), conventional medicine (CM), and both treatments according to the answers to “Are you currently taking any herbal or traditional remedy for your raised blood pressure?” (TM) and “In the past two weeks, have you taken any drugs (medication) for raised blood pressure prescribed by a doctor or other health worker?” (CM) [see Additional file 1 for the specific question to the other MRF].

### Statistical analysis

We conducted all statistical analyses using Stata version 15.0 (StataCorp, College Station, TX, USA). Firstly, we described the frequency for individuals with an MRF diagnosis who were not undergoing any type of treatment, those treated with only conventional medicine (CM), those using only traditional medicine (TM), and those combining conventional and traditional simultaneously according to sociodemographic, economic, and clinical characteristics. We compared the sociodemographic, economic and clinical characteristics using proportions and Fisher’s exact test for categorical variables, and the mean and Student’s t-test for continuous variables.

Secondly, to determine the frequency of TM use for the management of MRF and identify the socio-economic factors associated with its use, we described the proportion of individuals with an MRF who were taking TM, either alone or in combination with CM, among the total population who were taking some kind of treatment for their MRF. We used logistic regression to estimate Odds Ratios (ORs) with 95% confidence intervals and created a multivariable model using backward elimination considering all variables that were associated with the outcome variable (use of TM) in the univariate analysis with at least a p-value ≤ 0.1 and the sex variable due to the ingrained gender differences among the population. Missing data were excluded from the logistic regression analysis. We assessed multicollinearity using the correlation matrix and the Variance Inflation Factor (VIF).

### Ethics approval and consent to participate

The study protocol was reviewed by the ethical board at the Pontificia Universidad Católica de Ecuador (PUCE, reference 2019–27-MB). Participants were fully informed about the study's purpose, procedures, potential risks, benefits and the right to withdraw from the study at any time. All participants provided written informed consent.

## Results

### Study population

The study population, consisting of 602 participants, has a predominance of women, accounting for 68% of participants in both Esmeraldas (*N* = 186) and Quito (*N* = 224). In both settings, there are significant socioeconomic differences between men and women (Table 1). The mean age in Esmeraldas was 51 ± 16 years and in Quito 55 ± 16 years. Education levels in Esmeraldas are lower than in Quito, where the majority of participants have achieved secondary/higher education (67%). Ethnic predominance is characterised by Afro ethnicity in Esmeraldas and by Mestizo ethnicity in Quito. In Esmeraldas, half of the participants (*N* = 139, 57%) reported a monthly household income of less than $100, while in Quito, 59% (*N* = 155) reported a monthly household income of more than $375. In Esmeraldas, over two thirds lived in rural areas of the region (*N* = 176, 64%). People predominantly reported having only one of the MRF considered, with proportions of 59% in Esmeraldas (*N* = 161) and 67% in Quito (*N* = 229).

**Table 1:** Baseline characteristics of the study population

	Esmeraldas			Quito		
	Men	Women	*p*-value*	Men	Women	*p*-value*
	*N* (%)	*N* (%)		*N* (%)	*N* (%)	
**Age** (Mean ± SD)	57 ± 14	48 ± 16	**< 0.001**	57 ± 16	54 ± 15	0.201
**Education**^a^			0.217			0.092
No formal schooling	33 (37.5)	47 (25.4)		2 (1.9)	16 (7.1)	
Primary school	34 (38.6)	79 (42.7)		27 (26.0)	63 (28.1)	
Secondary school	17 (19.3)	49 (26.5)		40 (38.5)	92 (41.1)	
Higher education	4 (4.6)	10 (5.4)		35 (33.7)	53 (23.7)	
**Ethnicity**^b^			0.847			0.853
Afro	58 (65.9)	117 (62.9)		97 (94.2)	210 (94.6)	
Mestizo	26 (29.6)	61 (32.8)		1 (1.0)	1 (0.5)	
White				2 (1.9)	6 (2.7)	
Indigenous	4 (4.6)	8 (4.3)		3 (2.9)	5 (2.3)	
**Employment Status**			**< 0.001**			**0.017**
Unemployed	9 (10.2)	134 (72.0)		38 (36.5)	114 (50.9)	
Employed	79 (89.8)	52 (28.0)		66 (63.5)	110 (49.1)	
**Civil status**			0.259			**0.027**
Unpartnered	22 (25.0)	60 (32.3)		28 (27.9)	92 (41.1)	
Partnered	66 (75.0)	126 (67.7)		75 (72.1)	132 (58.9)	
**Household Earnings**^c^ Esmeraldas/Quito			0.101			**< 0.001**
≤$100 / ≤$375	40 (49.4)	99 (60.7)		22 (25.9)	88 (48.9)	
>$100 / >$375	41 (50.6)	64 (39.3)		63 (74.1)	92 (51.1)	
**Setting**			1.000			1.000
Rural	59 (67.1)	126 (67.7)		0 (0)	0 (0)	
Urban	29 (33.0)	60 (32.3)		104 (100)	224 (100)	
**Number of Risk Factors**			0.512			0.394
1	65 (73.9)	124 (66.7)		56 (53.8)	138 (61.6)	
2	20 (22.7)	53 (28.5)		37 (35.6)	65 (29.0)	
3	3 (3.4)	9 (4.8)		11 (10.6)	21 (9.4)	
**Hypertension**			0.137			1.000
No	17 (19.5)	53 (28.8)		50 (49.0)	108 (48.7)	
Yes	70 (80.5)	131 (71.2)		52 (51.0)	114 (51.3)	
**Hyperglycaemia**			1.000			0.895
No	44 (78.6)	104 (77.6)		47 (54.0)	95 (55.6)	
Yes	12 (21.4)	30 (22.4)		40 (46.0)	76 (44.4)	
**Hypercholesterol**			0.338			0.562
No	24 (42.9)	53 (35.6)		21 (22.8)	52 (26.9)	
Yes	32 (57.1)	96 (64.4)		71 (77.2)	141 (73.1)	
**Total**	88 (100)	186 (100)		104 (100)	224 (100)	

* p-value < 0.05 (t-student for age and Fisher’s exact test for the rest)

^a^1 not reported education level in Esmeraldas

^b^3 not reported ethnicity in Quito

^c^30 not reported household earnings in Esmeraldas, 63 in Quito

**Fig. 2:**
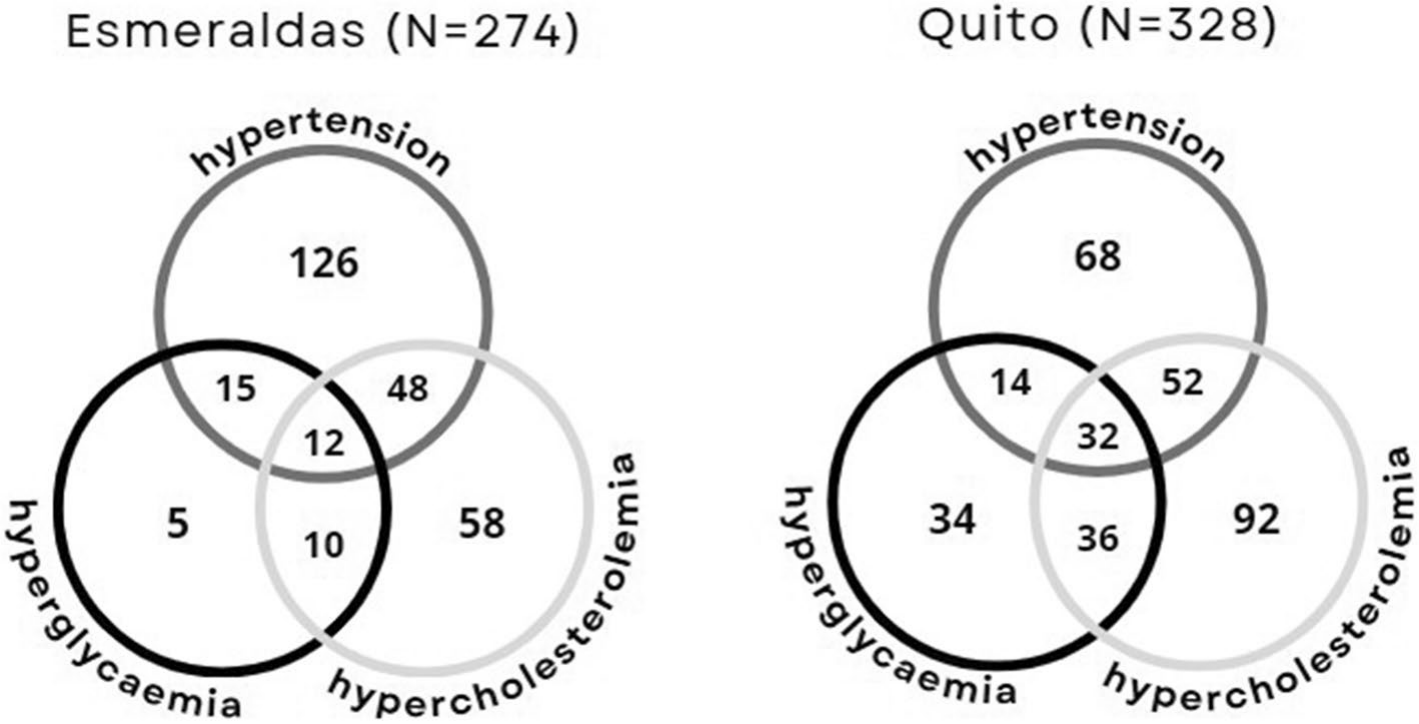
Overlapping of metabolic risk factors among the study population

In Esmeraldas, hypertension was the most prevalent MRF (201 individuals, 73%), followed by hypercholesterolemia (128, 47%) and hyperglycaemia (42, 15%). Conversely, in Quito, hypercholesterolemia stood out (212, 65%), followed by hypertension (166, 51%) and hyperglycaemia (116, 35%). A significant proportion of individuals had more than one MRF (85, 31% in Quito and 134, 40.9%) (Fig. 2).

### Type of treatment for the management of metabolic risk factors

Approximately 39.4% of the population in Esmeraldas used traditional medicine to control MRF (*N* = 108) as did 31.1% of the population in Quito (*N* = 90) (Table 2). Many of them were taking it in combination with conventional medicine; 63.9% (*N* = 102) in Esmeraldas and 44.1% (*N* = 45) in Quito. The percentage of people not taking any remedy to address their MRF was slightly higher in the capital (39.0%, *N* = 128) than in Esmeraldas (33.9%, *N* = 93). Individuals who did not take any treatments, or those that relied exclusively on traditional medicine tended to be younger, in both Quito and Esmeraldas (Table 2). Furthermore, individuals with more than one MRF were more likely to report use of conventional medicine, either alone or together with traditional medicine. Use of traditional medicine was more frequently observed among people with a higher level of education. While there are differences in household income levels in Esmeraldas, employment status and marital status stand out in Quito (Table 2).

**Table 2:** Type of treatment used according to sociodemographic, economic, and clinical characteristics in Esmeraldas and in Quito

Type of treatment (Tx)	Esmeraldas						Quito					
	No Tx	CM	TM	Both	Total	*p*-value*	No Tx	CM	TM	Both	Total	*p*-value*
**Sex**						0.670						0.079
Men	30 (34.1)	27 (30.7)	10 (11.4)	21 (23.9)	88 (100)		49 (47.1)	30 (28.8)	17 (16.4)	8 (7.7)	104 (100)	
Women	63 (33.9)	46 (24.7)	29 (15.6)	48 (25.8)	186 (100)		79 (35.3)	68 (30.4)	40 (17.9)	37 (16.5)	224 (100)	
**Age in years**, mean (SD)	43 (± 15)	59 (± 15)	43 (± 13)	57 (± 15)	51 (± 20)	**< 0.001**	47 (± 15)	64 (± 13)	53 (± 14)	63 (± 10)	55 (± 16)	**< 0.001**
**Education level**^a^						**0.008**						**< 0.001**
No formal schooling	21 (26.3)	17 (21.3)	14 (17.5)	28 (35.0)	80 (100)		3 (16.7)	11 (61.1)	1 (5.6)	3 (16.7)	18 (100)	
Primary school	43 (38.1)	38 (33.6)	8 (7.1)	24 (21.2)	113 (100)		25 (27.8)	40 (44.4)	13 (14.4)	12 (13.3)	90 (100)	
Secondary or higher	28 (35.0)	18 (22.5)	17 (21.3)	17 (21.3)	80 (100)		100 (45.5)	47 (21.4)	43 (19.5)	30 (13.6)	220 (100)	
**Ethnicity**^b^				11	21	0.180						0.801
Afro	52 (29.7)	44 (25.1)	26 (14.9)	53 (30.3)	175 (100)		121 (39.4)	90 (29.3)	54 (17.6)	42 (13.7)	307 (100)	
Mestizo	35 (40.2)	26 (29.9)	12 (13.8)	14 (16.1)	87 (100)		0 (0.0)	1 (50.0)	1 (50.0)	0 (0.0)	2 (100)	
White	-	-	-	-	-		3 (37.5)	2 (25.0)	1 (12.5)	2 (25.0)	8 (100)	
Indigenous	6 (50.0)	3 (25.0)	1 (8.3)	2 (16.7)	12 (100)		2 (25.0)	4 (50.0)	1 (12.5)	1 (12.5)	8 (100)	
**Employment status**						0.211						**0.004**
Unemployed	43 (30.1)	43 (30.1)	24 (16.8)	33 (23.1)	143 (100)		46 (30.3)	58 (38.2)	24 (15.8)	24 (15.8)	152 (100)	
Employed	50 (38.2)	30 (22.9)	15 (11.5)	36 (27.5)	131 (100)		82 (46.6)	40 (22.7)	33 (18.8)	21 (11.9)	176 (100)	
**Civil status**						0.153						**0.001**
Unpartnered	21 (25.6)	27 (32.9)	10 (12.2)	24 (29.3)	82 (100)		32 (26.5)	38 (31.4)	27 (22.3)	24 (19.8)	121 (100)	
Partnered	72 (37.5)	46 (24.0)	29 (15.1)	45 (23.4)	192 (100)		96 (46.4)	60 (29.0)	30 (14.5)	21 (10.1)	207 (100)	
**Household earnings**^c^ Esmeraldas/Quito						**0.020**						0.320
≤$100/≤$375	50 (36.0)	41 (29.5)	10 (7.2)	38 (27.3)	139 (100)		39 (35.5)	35 (31.8)	18 (16.4)	18 (16.4)	110 (100)	
>$100/>$375	33 (31.4)	25 (23.8)	22 (21.0)	25 (23.8)	105 (100)		69 (44.5)	41 (26.4)	28 (18.1)	17 (11.0)	155 (100)	
**Setting**						0.071						-
Rural	65 (35.1)	41 (22.2)	31 (16.8)	48 (26.0)	185 (100)		-	-	-	-	-	
Urban	28 (31.5)	32 (36.0)	8 (9.0)	21 (23.6)	89 (100)		-	-	-	-	-	
**Number of MRF**						**< 0.001**						**< 0.001**
1	82 (43.4)	45 (23.8)	27 (14.3)	35 (18.5)	189 (100)		96 (49.5)	48 (24.7)	37 (19.1)	13 (6.7)	194 (100)	
2	10 (13.7)	23 (31.5)	11 (15.1)	29 (39.7)	73 (100)		28 (27.5)	39 (38.2)	18 (17.6)	17 (16.7)	102 (100)	
3	1 (8.3)	5 (41.7)	1 (8.3)	5 (41.7)	12 (100)		4 (12.5)	11 (34.4)	2 (6.3)	15 (46.9)	32 (100)	
**Total**	93 (33.9)	73 (26.6)	39 (14.2)	69 (25.2)	274 (100)		128 (39.0)	98 (29.9)	57 (17.4)	45 (13.7)	328 (100)	

*Abbreviations: Tx *Treatment, *CM* Conventional Medication, *TM* Traditional Medicine

*p-value < 0.05 (t-student for age and Fisher’s exact test for the rest)

^a^1 not reported education level in Esmeraldas

^b^3 not reported ethnicity in Quito

^c^30 not reported household earnings in Esmeraldas, 63 in Quito

### Use of traditional medicine

Among people who treated their MRF, more than half used TM alone or in combination with CM (59.7%, *N* = 108 in Esmeraldas; 51.0%, *N* = 102 in Quito). In the population from Esmeraldas, we found statistically significant differences in TM use according to age, education level, ethnicity, and setting (Table 3). While the probability of using TM appears to be lower in urban setting compared to rural areas (OR: 0.47; 95%CI 0.25–0.88; p-value = 0.019), the association is lost in the regression model after controlling for age, education and ethnicity. After adjusting for sex, education level, ethnicity, employment, and setting, the frequency of TM use decreases with age by approximately 3% for each additional year (adjusted OR: 0.97; 95% CI 0.95–0.99). Compared to the mestizo ethnicity, individuals of afro ethnicity are twice as likely to use TM (adjusted OR: 2.13; 95%CI 1.02–4.45), while the indigenous ethnicity showed no differences. Those with no formal education and those with secondary or higher education are also more likely to use TM compared to primary education, but in the adjusted model, only the lack of formal education remains significant (adjusted OR: 3.76; 95%CI 1.59–8.88). Women have 2.02 times more likelihood of using TM, though it does not reach statistical significance (adjusted OR: 2.02; 95%CI 0.83–4.85; *p* = 0.117).

**Table 3:** Characteristics associated with traditional medicine (TM) use among people treating their metabolic risk factors in Esmeraldas

Variable	TM *N* (%)	Total treated (*N* (%)	*p*-value	OR	95%CI	*p*-value	aOR^a^	95%CI	*p*-value*
**Sex**			0.259						
Men	31 (53.5)	58 (100)		1			1		
Women	77 (62.6)	123 (100)		1.46	0.78–2.74	0.242	2.02	0.83–4.85	0.117
**Age in years**, mean (SD)	52 (± 16)	54 (± 16)	**0.003**	0.97	**0.95–0.99**	**0.004**	0.97	**0.95–0.99**	**0.022**
**Education**			**0.008**						
No formal schooling	42 (71.2)	59 (100)		2.93	**1.41–6.11**	**0.004**	3.76	**1.59–8.88**	**0.003**
Primary school	32 (45.7)	70 (100)		1			1		
Secondary school or higher	34 (65.4)	52 (100)		2.24	**1.07–4.70**	**0.032**	2.06	0.87–4.83	0.099
**Ethnicity**			0.177						
Mestizo	26 (50.0)	52 (100)		1			1		
Afro	79 (64.2)	123 (100)		1.8	0.93–3.46	**0.081**	2.13	**1.02–4.45**	**0.046**
Indigenous	3 (50.0)	6 (100)		1	0.19–5.42	1.000	0.71	0.11–4.46	0.716
**Employment status**			0.449						
Unemployed	57 (57.0)	100 (100)		1			1		
Employed	51 (63.0)	81 (100)		1.28	0.70–2.37	0.416	1.72	0.76–3.91	0.197
**Marital Status**			0.522						
Unpartnered	34 (55.7)	61 (100)		1					
Partnered	74 (61.7)	120 (100)		1.28	0.68–2.39	0.443			
**Household Earnings**^**a**^			0.152						
≤$100	48 (53.9)	89 (100)		1					
>$100	47 (65.3)	72 (100)		1.61	0.85–3.04	0.147			
**Setting**			**0.025**						
Rural	79 (65.8)	120 (100)		1			1		
Urban	29 (47.6)	61 (100)		0.47	**0.25–0.88**	**0.019**	0.68	0.31–1.49	0.336
**Number of Risk Factors**			0.773						
1	62 (57.9)	107 (100)		1					
2	40 (63.5)	63 (100)		1.26	0.67–2.40	0.476			
3	6 (54.5)	11 (100)		0.87	0.25–3.03	0.828			
**Total**	108 (59.7)	181 (100)							

*p-value < 0.05 (t-student for age and Fisher’s exact test for the rest) Adjusted by sex, age, education, ethnicity, employment and setting

^a^20 not reported household earnings

In the univariate analysis of Quito, significant differences in age, education level, and employment status are observed (Table 4). Similar to Esmeraldas, age was significantly associated to TM use with a decrease of 4% per year (OR 0.96, 95% CI 0.94–0.99, p-value: 0.001), although the association lost its statistical significance in the multivariable model. After adjusting for gender, age, and employment status, individuals with secondary education or higher were more likely to use TM to control their MRF (OR 2.04, 95% CI 1.03–3.90). The multicollinearity analysis indicates minimal correlation. In Esmeraldas, the highest correlation was − 0.5778 between sex and employment, with VIF values of 1.62 for employment and 1.60 for sex. In Quito, the highest correlation was − 0.2346 between employment and age, with a maximum VIF of 1.40 for age.

**Table 4:** Characteristics associated with traditional medicine (TM) use among people treating their metabolic risk factors in Quito

Variable	TM *N* (%)	Total treated *N* (%)	*p*-value	OR	95%CI	*p*-value	aOR	95%CI	*p*-value*
**Sex**			0.347						
Men	25 (45.5)	55 (100)		1			1		
Women	77 (53.1)	45 (100)		1.36	0.73–2.53	0.335	1.53	0.79–2.98	0.208
**Age in years**, mean (SD)	57 (± 14)	60 (± 14)	**< 0.001**	0.96	**0.94–0.99**	**0.001**	0.97	0.85-1.00	0.056
**Education**			**0.002**						
No formal schooling	4 (26.7)	15 (100)		0.58	0.17–2.03	0.395	0.58	0.16–2.09	0.408
Primary school	25 (38.5)	65 (100)		1			1		
Secondary school or higher	73 (60.8)	120 (100)		2.49	**1.34–4.62**	**0.004**	2.04	**1.03–3.90**	**0.041**
**Ethnicity**^**a**^			0.821						
Mestizo	96 (51.6)	186 (100)		1					
Afro	1 (50.0)	2 (100)		0.94	0.06–15.21	0.964			
White	3 (60.0)	5 (100)		1.41	0.23–8.61	0.712			
Indigenous	2 (33.3)	6 (100)		0.47	0.84–2.62	0.388			
**Employment Status**			0.091						
Unemployed	48 (45.3)	106 (100)		1			1		
Employed	54 (57.4)	94 (100)		1.63	**0.93–2.86**	**0.087**	1.28	0.70–2.34	0.416
**Marital Status**			0.120						
Unpartnered	51 (57.3)	89 (100)		1					
Partnered	51 (45.9)	111 (100)		0.63	0.36–1.11	0.111			
**Household Earnings**^**b**^			0.873						
≤$375	36 (50.7)	71 (100)		1					
>$375	45 (52.3)	86 (100)		1.01	0.57-2.00	0.840			
**Number of Risk Factors**			0.471						
1	50 (51.0)	98 (100)		1					
2	35 (47.3)	74 (100)		0.86	0.47–1.58	0.629			
3	17 (60.7)	28 (100)		1.48	0.63–3.49	0.366			
**Total**	**102 (51.0)**	**200 (100)**							

*p-value < 0.05 (t-student for age and Fisher’s exact test for the rest) Adjusted by sex, age, education and employment

^a^1 not reported ethnicity

^b^43 not reported household earnings

## Discussion

In our study, we observed that the use of TM to address MRF is quite widespread in the two health districts under investigation. Although some individuals reported not taking any treatment for their MRF, among those who were taking something, more than half included TM, either as monotherapy or in combination with CM. TM use in these settings appears to be associated with factors such as education, ethnicity and age, with more frequent use among younger populations. Surprisingly, we also found a high prevalence of individuals not taking any treatment, with this being more pronounced in Quito. This finding has significant implications for public health interventions, highlighting a critical need to address gaps in treatment adherence in both contexts taking socio-demographic characteristics into account, especially given the associated risk of comorbidities and adverse health outcomes of a poorly controlled diabetes.

In the case of Esmeraldas, and as seen in other low-middle income contexts [31], people without formal education were more likely to use TM. This was not the case in the urban setting of Quito. This discrepancy could be due to contextual differences and the higher education level in the capital. In both Quito and Esmeraldas, we found that those with higher levels of education were more likely to use this form of therapy compared to those with primary school education. Higher education often leads to increased access to information and increases individuals’ capacity for critical medical choices [32], which could influence the choice of TM to integrate it into self-care practices. For instance, Maidana et al. conducted a study on TM use for diabetes, where the majority of the population had high education levels [33]. A study conducted in 2019 in a rural area of ​​Ecuador that examined the use of TM to treat acute and chronic problems such as hyperglycaemia and hypertension showed very similar values ​​to our study for the rural population of Esmeraldas [21]. The impact of education in the use of TM should be further researched. In another study conducted in Malaysia comparing TM and CM for the control of cardiovascular risk factors, it was observed that CM was used more frequently, followed by the combination of CM and TM and finally TM alone. The results of TM application, both in isolation and in combination with CM, were similar to ours (31.7%) [34]. Comparison with other studies is difficult because they tend to analyse the use of TM without specifying whether it is used in conjunction with CM or as monotherapy.

Analysis of progress since the adoption of the World Health Organization’s first global strategy on TM clearly shows that the demand for traditional and complementary medicine (TCM) remains constant worldwide [9]. TCM is used not only to treat diseases, particularly chronic ones, but also to prevent disease, improve and maintain health, and has proven to be cost-effective for some governments. Although several studies show that conventional medicine is usually the therapy of choice for the treatment of chronic diseases [21, 34], traditional medicine is often used in the treatment of these diseases [35, 36]. However, it must be considered that many socio-economic and cultural factors, in addition to education, can determine whether one treatment is preferred over another [37, 38]. For this reason, it is fundamental to effectively integrate TM into the conventional healthcare system using a multifaceted approach [39] and taking the social determinants of health into account [40]. Increased investment in research and capacity building are essential to support evidence-based regulation and foster innovation in the herbal medicine sector. The recent lunch in July 2024 of the Ecuadorian Pharmacopoeia marks a significant step toward enhancing knowledge about traditional herbs and ensuring the safety and regulation of traditional medicine practices [41].

Our study has several limitations. It would have been interesting to conduct a comprehensive analysis of the type of treatment for each individual metabolic risk factor, but the sample was not large enough as it was originally selected to measure the prevalence of diabetes and other risk factors for non-communicable diseases in the selected health districts and not specifically to address the frequency of TM use to address risk factors, as we have done here. To address this limitation in part, we performed the main analysis with the combination of the three MRF allowing sufficient sample to describe TM use and explore some of the factors associated. However, it is still possible that insufficient sample size, may lead to us having insufficient statistical power to detect meaningful associations. Furthermore, it would have been beneficial to include additional questions to collect information regarding the reasons for choosing one therapy over another or the specific medications used, and include a qualitative component in the study to provide a more in-depth exploration of cultural and belief-related aspects, such as the specific traditional medicine practices. These limitations are linked to the fact that the current analysis was performed in a subset of the population surveys carried out as part of a larger project. However, it is important to point out that this fact also provides strengths to the analysis as the sample is representative of the general population in these areas, and the findings can be used to gauge the frequency of TM use at population level. Another limitation is the use of self-reported data, which may be subject to recall bias or social desirability bias, which could affect the reliability of the reported diagnoses and the extent of TM and CM use.

We report the data from two very different sociocultural regions of Ecuador. We did not conduct a joint analysis in order to avoid losing sensitivity in our analysis of variables like ethnicity, household income or urban-rural setting. For example, the urbanised environment in Esmeraldas, is very different from Quito, although both are classified as “urban settings” in the analysis.

## Conclusions

Overall, the results obtained highlight the widespread use of TM in the different sociocultural settings studied, and the need to integrate the use of TM based on local beliefs in the management of MRF in order to provide high quality intercultural healthcare that reduces health disparities. It is often used in combination with conventional medicine, and research is needed to address safety concerns over potential interactions with conventional medicines. It is imperative for health professionals, authorities, and the general population to collaborate in order to achieve a more accessible and intercultural approach to the management of cardiovascular risk, where decisions are shared and based in evidence.

## Supplementary Information

Additional file 1.

## Data Availability

The datasets generated and analysed during the current study are available in the Zenodo repository, with the DOI: 10.5281/zenodo.10853344.

## Abbreviations

aOR: adjusted Odds Ratio
CECOMET: Centre of Community Epidemiology and Tropical Medicine
CM: Conventional Medication
CVD: Cardiovascular Diseases
MAIS-FCI: Manual of the Comprehensive Care Model of the National Family and Community Health System
MRF: Metabolic Risk Factors
NCDs: Non-Communicable Diseases
NHS: National Health System
OR: Odds Ratio
TCM: Traditional and Complementary Medicine
TM: Traditional Medicine
Tx: Treatment
VIF: Variance Inflation Factor
WHO: World Health Organization
