# Supplementary material for: Prevalence and factors associated with the use of traditional medicine in individuals with hypercholesterolemia, hyperglycaemia, and arterial hypertension in Ecuador: results from a population-based study in two health districts
Source: BMC Complement Med Ther. 2024 Oct 7;24:363. doi: 10.1186/s12906-024-04666-0 (PMC11460002; doi:10.1186/s12906-024-04666-0)
Supplement: Supplementary file 1 — Additional file 1. [file 12906_2024_4666_MOESM1_ESM.docx]

**Additional file 1.** Questions for collecting information about metabolic risk factors and their treatment.

***Blood pressure:***

- Has a doctor or another healthcare provider ever told you that you have high blood pressure or hypertension?
- In the last two weeks, have you taken any medication (medicine) to treat high blood pressure, prescribed by a doctor or another healthcare provider?
- Have you ever consulted with a traditional healer for high blood pressure or hypertension?
- Are you currently taking any herbal or traditional remedy for high blood pressure?

***Glycaemia:***

- Has a doctor or another healthcare provider ever told you that you have high blood pressure or hypertension?
- In the last two weeks, have you taken any medication (medicine) to treat high blood pressure, prescribed by a doctor or another healthcare provider?
- Have you ever consulted with a traditional healer for high blood pressure or hypertension?
- Are you currently taking any herbal or traditional remedy for high blood pressure?

***Cholesterolemia***

- Has a doctor or another healthcare provider ever told you that you have elevated blood cholesterol?
- In the last two weeks, have you taken any oral medication to treat elevated cholesterol, prescribed by a doctor or another healthcare provider?
- Have you ever consulted with a traditional healer for elevated cholesterol?
- Are you currently taking any herbal or traditional remedy for elevated cholesterol?
